# Arabidopsis aldehyde dehydrogenase 10 family members confer salt tolerance through putrescine-derived 4-aminobutyrate (GABA) production

**DOI:** 10.1038/srep35115

**Published:** 2016-10-11

**Authors:** Adel Zarei, Christopher P. Trobacher, Barry J. Shelp

**Affiliations:** 1 Department of Plant Agriculture, University of Guelph, Guelph, Ontario, N1G 2W, Canada

## Abstract

Polyamines represent a potential source of 4-aminobutyrate (GABA) in plants exposed to abiotic stress. Terminal catabolism of putrescine in *Arabidopsis thaliana* involves amine oxidase and the production of 4-aminobutanal, which is a substrate for NAD^+^-dependent aminoaldehyde dehydrogenase (AMADH). Here, two AMADH homologs were chosen (*At*ALDH10A8 and *At*ALDH10A9) as candidates for encoding 4-aminobutanal dehydrogenase activity for GABA synthesis. The two genes were cloned and soluble recombinant proteins were produced in *Escherichia coli*. The pH optima for activity and catalytic efficiency of recombinant *At*ALDH10A8 with 3-aminopropanal as substrate was 10.5 and 8.5, respectively, whereas the optima for *At*ALDH10A9 were approximately 9.5. Maximal activity and catalytic efficiency were obtained with NAD^+^ and 3-aminopropanal, followed by 4-aminobutanal; negligible activity was obtained with betaine aldehyde. NAD^+^ reduction was accompanied by the production of GABA and β-alanine, respectively, with 4-aminobutanal and 3-aminopropanal as substrates. Transient co-expression systems using Arabidopsis cell suspension protoplasts or onion epidermal cells and several organelle markers revealed that *At*ALDH10A9 was peroxisomal, but *At*ALDH10A8 was cytosolic, although the N-terminal 140 amino acid sequence of *At*ALDH10A8 localized to the plastid. Root growth of single loss-of-function mutants was more sensitive to salinity than wild-type plants, and this was accompanied by reduced GABA accumulation.

The non-proteinogenic amino acid 4-aminobutyrate (GABA) accumulates in plants in response to various abiotic stresses such as chilling, drought and salinity^1^,^2^. In dicotyledonous plants, it can be biosynthesized via two distinct pathways: from glutamate via pH- and calmodulin-dependent glutamate decarboxylase activity^3^,^4^; and, from terminal oxidation of the diamine putrescine and the polyamine spermidine via the action of copper-containing amine oxidase (CuAO, EC 1.4.3.22) and FAD-dependent polyamine oxidase (PAO, E.C. 1.5.3.6), respectively^5^,^6^,^7^,^8^. CuAOs can catalyze the conversion of 1,3-diaminopropane to 3-aminopropanal (APAL), as well as putrescine to 4-aminobutanal (ABAL). Oxidation of ABAL and APAL is often attributed to the activity of NAD^+^-dependent aminoaldehyde dehydrogenases (AMADH, EC 1.2.1.19)^5^,^9^, leading to GABA and β-alanine biosynthesis, respectively. Plant AMADHs, which can catalyze the oxidation of ω-aminoaldehydes to the corresponding ω-amino acids, are included in the aldehyde dehydrogenase 10 family^10^.

Two putative *AMADH* genes have been identified from *Arabidopsis, AtALDH10A8* and *AtALDH10A9*^11^, but their characterization is incomplete. The available evidence suggests that recombinant *At*ALDH10A9 reduces NAD^+^ in the presence of betaine aldehyde (BAL), 4-aminobutanal (ABAL) or 3-aminopropanal (APAL), whereas the biochemical properties of *At*ALDH10A8 have not yet been investigated. The N-termini of *At*ALDH10A8 and *At*ALDH10A9 are localized to the leucoplast and peroxisome, respectively. Both *AtALDH10A8* and *AtALDH10A9* genes are expressed constitutively in Arabidopsis^12^, although they have been reported to be weakly induced by salinity and dehydration^11^. Other research has shown that efficient BAL oxidation by the ALDH10 family of plant proteins apparently requires that the Ile residue position 444 (*At*ALDH10A8 numbering, residue 441 for *Spinacia oleracea* BADH numbering) is occupied by Ala or Cys^13^,^14^,^15^. Furthermore, two apple AMADHs (*MdAMADH2 and MdAMADH1* designated here as *MdALDH10A8 and MdALDH10A9*) are active with NAD^+^ and APAL or ABAL, resulting in the production of β-alanine and GABA, respectively, whereas BAL is a poor substrate^16^. Notably, both apple and Arabidopsis members of the ALDH10 family of proteins possess Ile at position 444^16^.

In this paper, we revisited the previously characterized *AtALDH10* genes from Arabidopsis. They were both successfully expressed in *Escherichia coli* and the resulting recombinant proteins were biochemically characterized. APAL and ABAL, but not BAL, were effective substrates for the production of β-alanine and GABA, respectively. Transient expression of green fluorescent protein (GFP) fusions in Arabidopsis protoplasts or onion epidermal cells confirmed that *At*ALDH10A9 is peroxisomal, whereas *At*ALDH10A8 appeared to be cytosolic, although the N-terminal 140 amino acid sequence of *At*ALDH10A8 localized to the plastid. Finally, we demonstrated that *ataldh10A8* and *ataldh10A9* T-DNA-insertion mutants have lower levels of GABA than wild-type (WT) plants in response to salinity treatment and are more prone to salinity stress.

## Results

### Arabidopsis ALDH10 members displayed catalytic activity at different pH optima

The recombinant His-tagged proteins were purified to homogeneity, as indicated by Coomassie blue staining and immunoblot analysis of the soluble and affinity-purified fractions (Supplementary Fig. S1). They retained their enzymatic activity for several months after ammonium sulphate precipitation and storage at −80 °C. Enzyme activity was initially determined as the production of NADH during the oxidation of APAL. Preliminary characterization indicated that initial rates of *At*ALADH10A8 and *At*ALDH10A9 activities display sharp pH optima at 10.5 and 9.5–9.7, respectively, at a saturating level of ABAL (Supplementary Fig. S2A) and subsaturating level of APAL (Supplementary Fig. S2B). Both enzymes retained approximately 20% of their maximal substrate-limited, APAL-dependent activity at pH 7.5 and were inactive at pH 6.5 (Supplementary Fig. S2B). Further study of enzymatic activities near the pH optima revealed that *At*ALDH10A9 displayed a slightly higher *V*_max_ and catalytic efficiency at pH 9.5 than pH 8.5, and *At*ALDH108 a slightly lower *V*_max_ at pH 8.5 than pH 9.5 and 10.5, but the catalytic efficiency was slightly higher at pH 8.5 (Supplementary Fig. S3, Supplementary Table S1).

### *At*ALDH10A8 and *At*ALDH10A9 effectively utilized APAL and ABAL as substrates, but not BAL

The concentrations of *At*ALDH10A8 and *At*ALDH10A9 protein were adjusted to be within the linear range for both APAL (10 nM) and ABAL (50 nM) in all enzymatic assays. BAL-dependent activity was not detectable with 10 nM protein concentration and the activity with 100 nM protein was less than 0.5% of that for APAL; therefore, BAL was not included in kinetic studies. The maximal APAL-dependent activity of *At*ALDH10A8 with 1 mM NADP^+^, presumably saturating, was 40% of that with NAD^+^, whereas the activity of *At*ALDH10A9 was only 10–15% (data not shown).

Fitting of the NAD^+^-dependent activities of *At*ALDH10A8 at the optimum pH to a Michaelis-Menten equation that considers substrate inhibition revealed that both APAL and ABAL displayed partial substrate inhibition (as indicated by *K*_is_); however, it was ten times stronger for APAL than ABAL (Table 1; Supplementary Fig. S4). The corresponding activities for *At*ALDH10A9 had the same trends, but the inhibition was much less than for *At*ALDH10A8. *At*ALDH10A8 and *At*ALDH10A9 exhibited two and 25 times stronger affinity and 20 and 40 times higher catalytic efficiency, respectively, for APAL than ABAL (Table 1). When APAL and ABAL were provided at subsaturating levels to limit substrate inhibition and allow for measureable activities, there was a three- to seven-fold range in affinity for NAD^+^ between the enzymes, with *At*ALDH10A8 generally displaying the stronger affinity. However, the catalytic efficiencies for NAD^+^ with APAL or ABAL were similar across the two enzymes (Table 1).

### *At*ALDH10A8 and *At*ALDH10A9 catalyzed the synthesis of GABA and β-alanine

**Figure 1:**
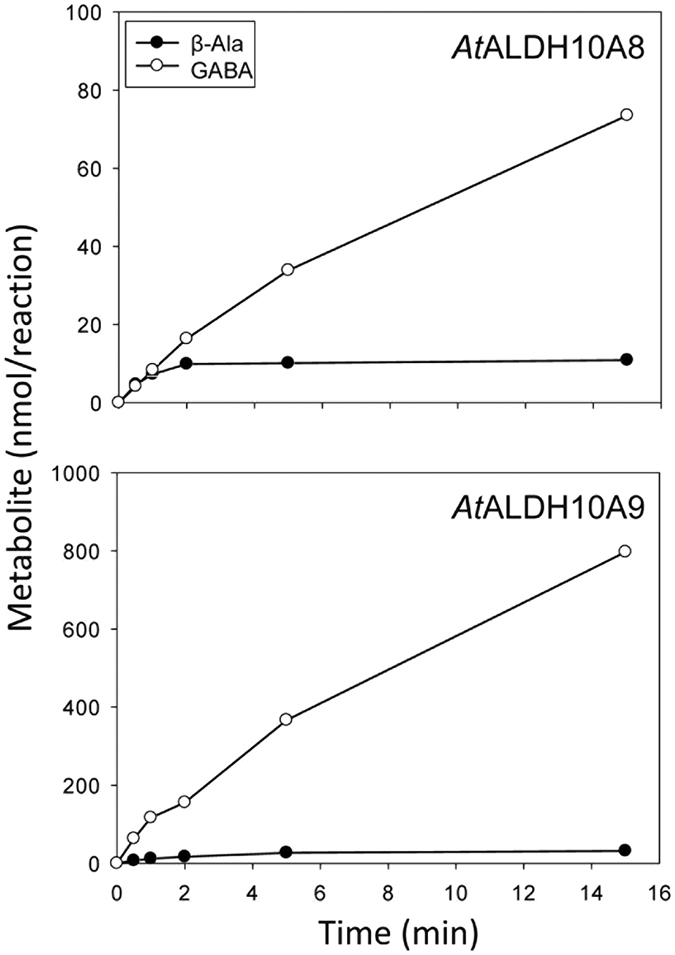
*In vitro* production of GABA or β-alanine by recombinant *At*ALDH10A8 and *At*ALDH10A9. For assay of ABAL-dependent activities, 50 nM protein was used with 0.1 mM NAD^+^ and 50 μM ABAL for *At*ALDH10A8, or 0.5 mM NAD^+^ and 800 μM ABAL for *At*ALDH10A9; for assay of APAL-dependent activities, the reactions contained 10 nM protein with 0.1 mM NAD^+^ and 20 μM APAL for *At*ALDH10A8 or 0.5 mM NAD^+^ and 50 μM APAL for *At*ALDH10A9. Data represent the mean of three technical replicates from a typical enzyme preparation. Closed and open circles indicate GABA and β-alanine concentration, respectively. The estimated rates of GABA production at 30 s were 0.59 and 8.9 μmol min^−1 ^mg^−1^ protein for *At*ALDH10A8 and *At*ALDH10A9, respectively. The corresponding rates of β-alanine production were 3.3 and 4.8 μmol min^−1 ^mg^−1^ protein, respectively. Substrate conversion after 15 min was 20–29% and 11–12%, respectively, for the ABAL- and APAL-dependent reactions. The assays were performed as 5-mL reactions.

To confirm the biochemical functions of *At*ALDH10A8 and *At*ALDH10A9, ABAL and APAL were individually supplied to the recombinant proteins *in vitro* at concentrations resulting in apparent *V*_max_ (Supplementary Fig. S4). The NAD^+^ concentration was adjusted to 0.1 mM and 0.5 mM for *At*ALDH10A8 and *At*ALDH10A9, respectively. The estimated rates of GABA production at 30 s were 0.59 and 8.9 μmol min^−1 ^mg^−1^ protein for *At*ALDH10A8 and *At*ALDH10A9, respectively, while the corresponding rates of β-alanine production were 3.3 and 4.8 μmol min^−1 ^mg^−1^ protein (Fig. 1). Furthermore, the APAL-dependent reactions ceased within 2–5 min, and substrate conversion after 15 min was 20–29% and 11–12%, respectively, for the ABAL- and APAL-dependent reactions.

### *At*LADH10A8 and *At*ALDH10A9 localized to peroxisome and plastid

**Figure 2:**
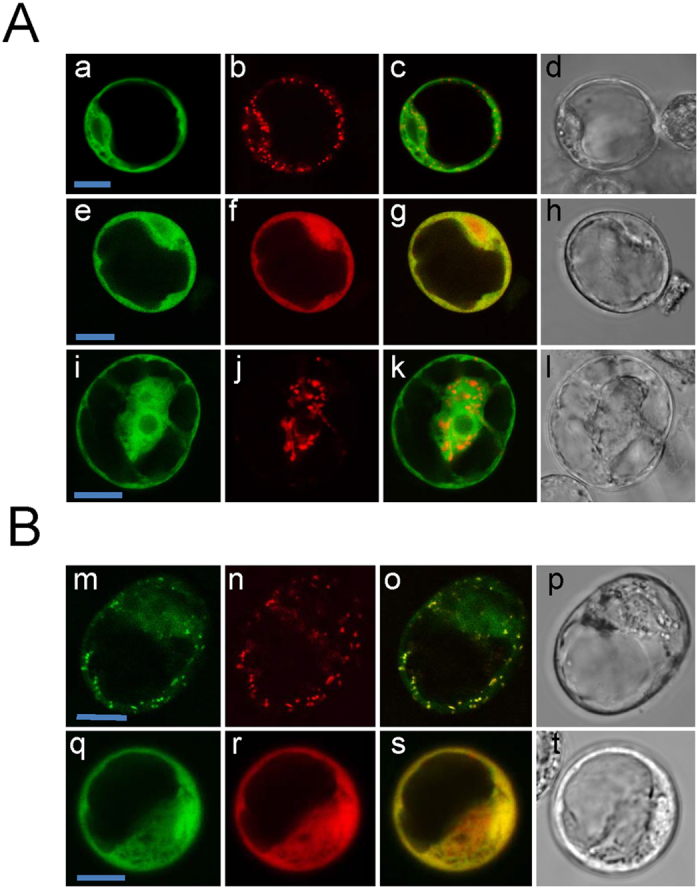
Subcellular localization of *At*ALDH10A8 and *At*ALDH10A9 fusion proteins in Arabidopsis cell suspension protoplasts. Confocal laser scanning microscopic images of protoplasts transiently co-expressing GFP fusion protein and peroxisomal, cytosolic or plastid markers. (**A**) GFP-*At*ALDH10A8 (a,e) was co-expressed with a mCherry peroxisome marker (b) or RFP cytosol (f). *At*ALDH10A8-GFP (i) was co-expressed with RFP plastid marker (j). (**B**) GFP-*At*ALDH10A9 (m) and *At*ALDH10A9-GFP (q) were co-expressed with mCherry peroxisomal marker (n) or a RFP cytosol marker (r). Merged images of the green and red signals appear yellow (c,g,k,o,s). Cell morphology was observed with transmitted light microscopy (d,h,i,p,t). Images were taken 16 h after protoplast transformation. Scale bars = 10 μm.

To examine the subcellular localization of Arabidopsis ALDH10s, Arabidopsis protoplasts were transformed with GFP-fusion constructs using polyethylene glycol. Organellar markers such as mCherry peroxisomal, Red Fluorescent Protein (RFP) cytosolic and RFP plastidial were employed. The subcellular locations of the GFP-tagged ALDH10s were determined by confocal laser-scanning microscopy. In all cases, both C- and N-terminal GFP fusion constructs were generated to exclude the possibility that localization was dependent on the position of the GFP tag. GFP-*At*ALDH10A8 and *At*ALDH10A8-GFP fusion proteins displayed a diffuse signal in the cytosol and did not co-localize with mCherry peroxisomal and RFP plastidial markers (Fig. 2A). In contrast, GFP-*At*ALDH10A9 strongly co-localized with mCherry peroxisomal (Fig. 2B) and *At*ALDH10A9-GFP, like peroxisomal *Md*ALDH10A8-GFP C-terminal fusion proteins^16^, localized to the cytosol (Fig. 2B).

**Figure 3:**
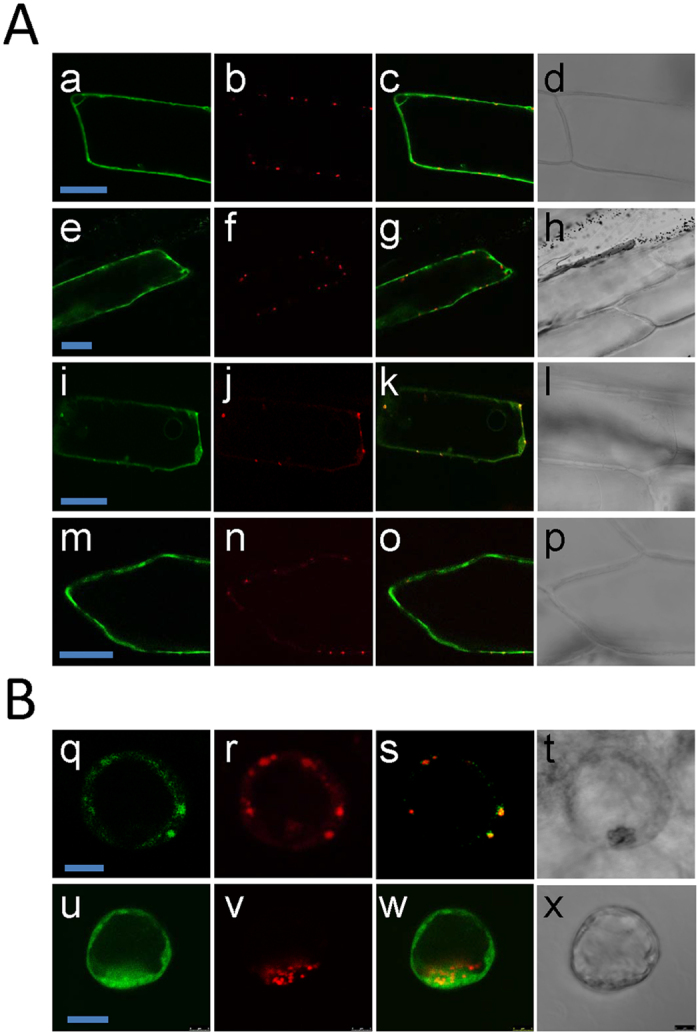
Subcellular localization of *At*ALDH10A8 and C-terminal deletion derivatives of *At*ALDH10A8 in onion epidermal cells and Arabidopsis cell suspension protoplasts. (**A**) Confocal laser scanning microscopic images of onion epidermal cells transiently co-expressing *At*ALDH10A8-GFP (a) GFP-*At*ALDH10A8 (e) *At*ALDH10A8140-GFP (i) or GFP-*At*ALDH10A8140 with RFP plastid marker (b, f, j, n). Bars represent 40 μm. (**B**) Confocal laser scanning microscopic images of Arabidopsis cell suspension protoplasts expressing *At*ALDH10A8140-GFP (q) or GFP-*At*ALDH10A8 140 (u) with RFP plastid marker (r,v). Cells were incubated for 16 h and then processed for microscopy in both cases. Corresponding green and red merged images appear yellow in the third column (c,g,k,o,s,w) and transmitted light microscopy images are at the right (d,h,l,p,t,x). Scale bars = 10 μm.

To establish whether localization of *At*ALDH10A8 in the cytosol is a peculiarity of Arabidopsis protoplasts or a more general phenomenon, onion epidermal cells were transformed using biolistic bombardment. Expressed GFP-*At*ALDH10A8 and *At*ALDH10A8-GFP fusion proteins resided in the cytosol of epidermal cells up to 24 h after transformation (Fig. 3A). Unlike *At*ALDH10A9, *At*ALDH10A8 does not possess a C-terminal peroxisomal targeting signal 1 (PTS1) motif, and WoLF PSORT online software (http://www.genscript.com/wolf-psort.html) predicts the protein to be localized in the chloroplast. Consequently, constructs containing the N-terminal 140 amino acid sequence of *At*ALDH10A8 were fused to GFP at the N- and C-termini (GFP-*At*ALDH10A8-140 and *At*ALDH10A8-140-GFP). Notably, the green fluorescence signal of the expressed *At*ALDH10A8-140-GFP fusion protein coincided with the RFP plastidial marker in both onion epidermal cells (Fig. 3Ai–l) and Arabidopsis protoplasts (Fig. 3B, q–t), although GFP-*At*ALDH10A8-140 still resided in the cytosol of both cell types (Fig. 3A, m, n, o, p and Fig. 3B, u–x). To exclude the possibility that cleavage of *At*ALDH10A8-GFP occurred with the release of the GFP into the cytosol, *At*ALDH10A8-GFP was co-expressed with GFP in Arabidopsis protoplasts. Total protein was extracted and subjected to Coomassie Brilliant Blue staining and immunoblot analysis using an anti-GFP antibody (Supplementary Fig. S5). The results revealed the presence of intact *At*ALDH10A8-GFP. To assess whether fluorescent bodies observed in transformed protoplasts expressing the *At*ALDH10A8-GFP fusion protein might be subject to proteasome-mediated degradation, transformed protoplasts were treated with a proteasomal inhibitor, MG132, for 4 h after 12 h incubation. Immunoblot analysis using an anti-GFP antibody revealed that the total amount of *At*ALDH10A8-GFP was not increased by MG132 (Supplementary Fig. S5).

### Arabidopsis *aldh10a8* and *aldh10a9* knockout mutants were susceptible to salinity

**Figure 4:**
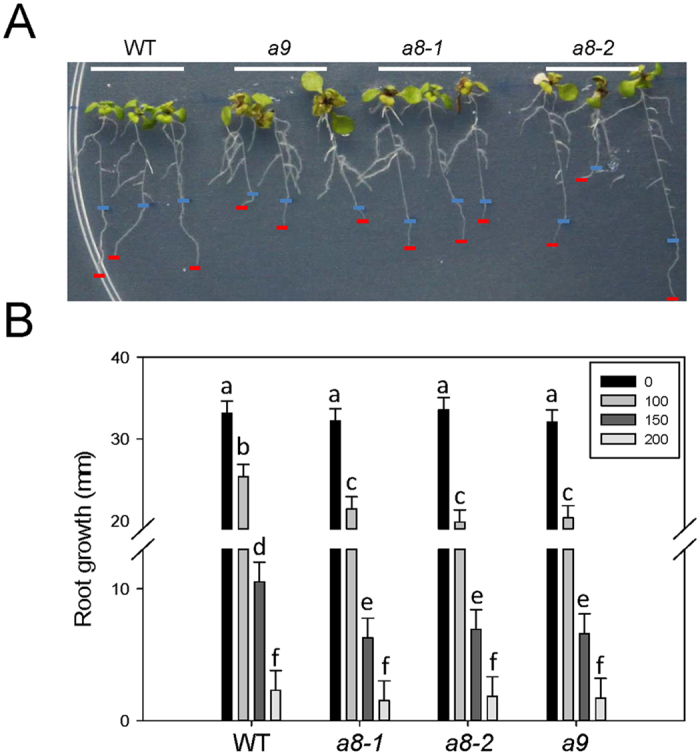
Salinity-induced phenotype of *ataldh10a8* and *at10aldh109* mutants. (**A**) Wild-type (WT) plants and *ataldh10A8 (A8-1* and *A8-2*) or *ataldh10A9 (A9*) were grown on half strength MS medium for 1 week and then transferred to the same medium containing 150 mM NaCl and grown for another 4 d before being photographed. The length of the root segment between the blue and red bars indicate root growth in the later stage. (**B**) Quantification of primary root growth on half strength MS medium containing different levels of salt stress. One-week-old seedlings of WT and *A8-1, A8-2* and *A9* T-DNA knockout mutants were transferred to new agar plates and grown for 4 d. Results are the least squared means ± S.E. of measurements from three plates (three plants per plate) estimated following two-way analysis of variance (ANOVA) performed in PROC MIXED procedure in SAS with P = 0.05 as the significance threshold. Bars sharing the same letter are not significantly different. Numbers in the box represent the concentrations of NaCl in mM.

To investigate the role of *At*ALDH10A8 and *At*ALDH10A9 genes in the stress response, homozygous T-DNA knockout lines (*ataldh10a8-1, ataldh10a8-2, ataldh10a9*) were screened and selected for further experimentation as shown in Supplementary Fig. S6. All three mutants had a phenotype similar to WT plants grown on agar or in soil under control conditions. The addition of 150 mM NaCl to half strength Murashige and Skoog (MS) medium caused a severe phenotype in the mutants grown on agar, including the appearance of necrotic lesions and purpling on leaves and inhibition of root growth, although roots seemed to be more sensitive than shoots (Fig. 4A). The root growth of all mutants grown on agar appeared to be oversensitive in medium containing 100 or 150 mM NaCl (Fig. 4B); for example, at 150 mM NaCl root growth was reduced by approximately 50% compared to 29% in the WT.

### Arabidopsis *aldh10a8* and *aldh10a9* knockout mutants accumulated less GABA in response to salinity

**Figure 5:**
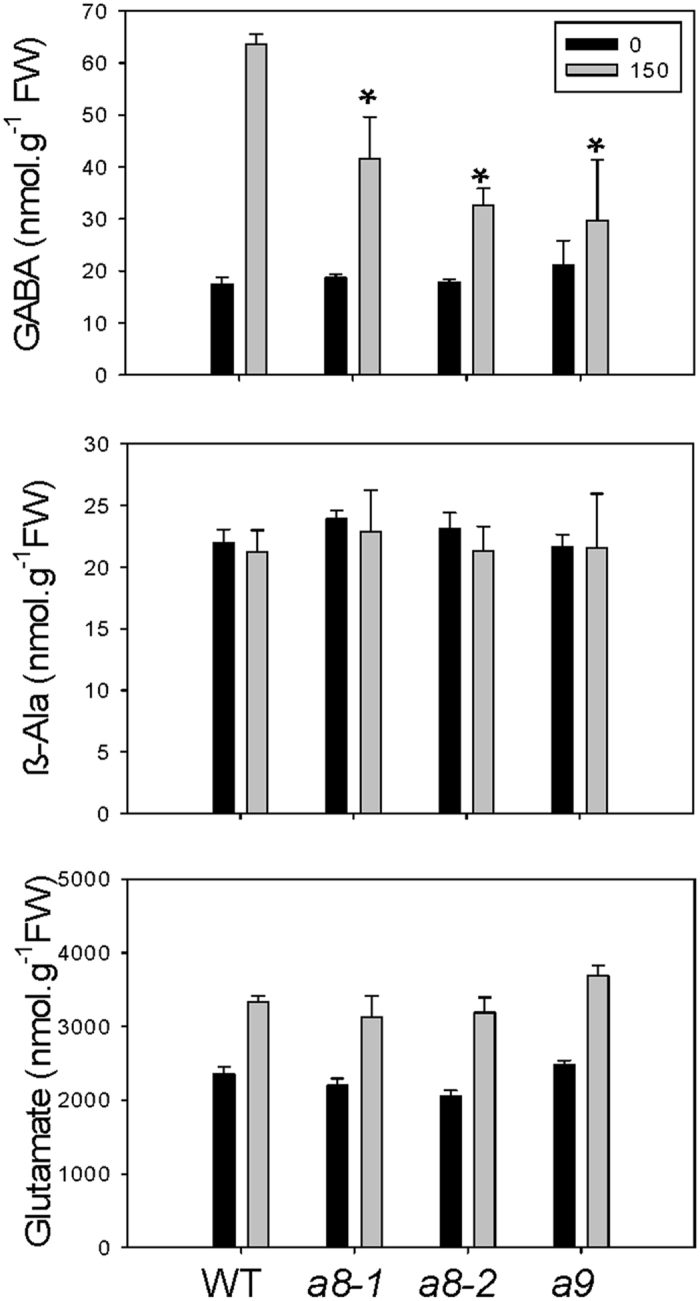
Salinity-induced chemotype of *ataldh10a8* and *ataldh10a9* mutants. Plants were watered with 150 mM NaCl as indicated in the Materials and Methods so that the plants were briefly submerged. Plant shoots were harvested 48 h after onset of salt stress. Each datum represents the mean (±SE) of three biological replicates. Metabolite concentrations are presented as nmol/g FW. Asterisk indicate significant differences between the mutants and the WT (P < 0.05, Student’s t-test) under salt stress. Numbers in the box represent the concentrations of NaCl in mM.

To evaluate the contribution of polyamine catabolism to GABA and β-alanine production in salinity-stressed plants, 4-week-old potted plants at the vegetative stage were treated with or without 150 mM NaCl for 2 d. The concentrations of GABA and β-alanine were similar in WT and *ataldh10* mutants under control conditions (Fig. 5A,B). Notably, the GABA concentration in untreated WT plants (20 nmol g^−1^ FW) was induced by approximately three-fold with salinity. This accumulation was reduced by approximately 50% in the mutant lines. There was no significant difference in GABA concentrations among the mutants. In contrast, the β-alanine concentrations did not change with salinity, and were similar between WT and the mutants (Fig. 5). The concentrations of glutamate, a precursor for GABA, were increased by approximately 30% with salinity in both WT and mutants (Fig. 5).

## Discussion

The biochemical properties of two putative Arabidopsis AMADHs, *At*ALDH10A8 and *At*ALDH10A9, were characterized in the present study. To our knowledge, the biochemical characterization of *At*ALDH10A8 has not been reported elsewhere. Based on catalytic efficiency, the pH optima for *At*ALDH10A9 and *At*ALDH10A8 appeared to be 9.5 and 8.5, respectively (Supplementary Table S1), whereas based on relative activity with both saturating and substaurating substrate, the pH optima were 9.5–9.7 and 10.5, respectively (Supplementary Fig. S1). Our findings are consistent with the pH optima of characterized plant AMADHs (i.e., apple AMADH: *Md*ALDH10A8 and *Md*ALDH10A9; tomato AMADH: *Sl*AMADH1, *Sl*AMADH2; corn AMADH: *Zm*AMADH1a, *Zm*AMADH1b, *Zm*AMADH2; spinach AMADH: *So*BADH; barley AMADH: *Hv*AMADH1, *Hv*AMADH2; and pea AMADH: *Ps*AMADH1, *Ps*AMADH2), which range between pH 8 and pH 10.2^13^,^16^,^17^,^18^,^19^. For example, the *Ps*AMADH1 and *Ps*AMADH2 paralogs display sharp pH optima of approximately 9.7 and 10.2, respectively, with saturating APAL^19^. Kopečný *et al*.^13^ have proposed that the thiolate of catalytic Cys294 is preserved when pH is significantly above 8, thereby accelerating nucleophilic attack. Since catalytic efficiency is likely to be more relevant *in vivo* wherein substrate saturation is never achieved, pHs 9.5 and 8.5, respectively, were chosen to further biochemically characterize *At*ALDH10A9 and *At*ALDH10A8. Notably, the pH optima of *At*ALDH10A9 and *At*ALDH10A8 are in good agreement with the pH of the cellular compartment in which they reside: the pHs of peroxisomes and chloroplasts are strongly alkaline and moderately alkaline, respectively^20^,^21^.

The Arabidopsis ALDH10As studied here favour NAD^+^ as the cofactor over NADP^+^ with APAL. The Glu188 residue (*At*ALDH10A8 numbering) is apparently responsible for this preference^22^. The Arabidopsis ALDH10As also had negligible activities with BAL, findings similar to those previously reported for *Zm*AMADH1b, *Zm*AMADH2, *Sl*AMADH2, *Ps*AMADHs, *Md*ALDH10A8 and *Md*ALDH10A9^13^,^16^. Notably, BAL oxidation seems to be dependent on the presence of Ala or Cys at position 444 (*At*ALDH10A8 numbering)^14^,^15^. Surprisingly, Missihoun *et al*.^11^ reported that *At*ALDH10A9 has higher catalytic efficiency with BAL than ABAL and APAL, although affinity for these three substrates is in the low millimolar range, rather than the micromolar range as reported here (Table 1). An explanation for the discrepancy between the two studies is not obvious; however, it is clear that our kinetic characterization of the homogeneous *At*ALDH10A preparations was conducted using optimized assay conditions (i.e., pH, concentration of desalted protein in the linear range, initial rates were determined, four-five substrate concentrations above and below the *K*_m_, kinetic parameters were estimated from three independent biological preparations; ABAL and APAL were prepared fresh daily). Furthermore, we accounted for the presence of substrate inhibition, which was particularly strong for *At*ALDH10A8, and demonstrated that both *At*ALDH10A8 and *At*ALDH10A9 possessed a higher preference for APAL than ABAL. These properties are typical of some previously reported AMADHs^13^,^15^,^16^,^23^.

Evidence was also provided for the stoichiometric production of NADH and GABA or β-alanine, respectively, when ABAL or APAL were utilized as substrates (Fig. 1). Despite no efforts to optimize *in vitro* assays for metabolite production, the initial rates were of the same order of magnitude or only slightly lower than the apparent *V*_max_ values obtained from measurements of substrate-dependent NADH production (Table 1). However, the conversion of substrate for the APAL-dependent *At*ALDH10A8 and *At*ALDH10A9 activities ceased after a brief period (Fig. 1), indicating that both enzymes became inactivated as the reaction proceeded. Similar observations have been reported for betaine aldehyde dehydrogenases (BADH; EC 1.2.1.8) from *Amaranthus hypochondriacus* and *Pseudomonas aeruginosa*^24^ and for APAL-dependent activities of *Md*ALDH10A8 and *Md*ALDH10A9^16^. Muñoz-Clares *et al*.^24^ have suggested that uncompetitive inhibition by the reduced pyridine dinucleotide product could explain these findings, and constitute another way to increase the effectiveness of reduced dinucleotides in inhibition. If so, it is unclear why the ABAL-dependent reactions were not similarly inactivated (Fig. 1 and ref. 16).

Amino acid sequence analysis has revealed that *At*ALDH10A9, like *Os*AMADH1, *Os*AMADH2, *Zm*AMADH1a, *Zm*AMADH1b, *Zm*AMADH2, *Hv*AMADH1, *Ps*AMADH1, *Ps*AMADH2, *Md*ALDH10A9 and *Sl*AMADH2, possess a C-terminal canonical peroxisomal targeting signal 1 (PTS1, SKL), whereas *At*ALDH10A8, *Hv*AMADH2, *So*BADH and *Sl*AMADH1 do not^16^. Missihoun *et al*.^11^ have reported that *At*ALDH10A8 localizes to the leucoplast, whereas *Hv*AMADH2 and *So*BADH are cytosolic and plastidial, respectively^17^,^18^. The subcellular location of *Sl*AMADH1 has not been studied. Here, the subcellular localization of the *At*ALDH10s was studied using Arabidopsis cell suspension protoplasts and onion epidermal cells (Figs 2 and 3), which provide two reliable transient expression systems for protein targeting. The peroxisomal localization of *At*ALDH10A9 was confirmed^11^, but *At*ALDH10A8 appeared to be cytosolic (Fig. 2). Missihoun *et al*.^11^ has also reported that *At*ALDH10A8 is cytosolic; however, its N-terminal portion (1–140 aa fused to GFP) apparently targets the leucoplast, rather than the chloroplast, although organelle markers were not used.

In the present study, the functionality of the predicted plastid targeting peptide in *At*ALDH10A8 was investigated with GFP N- and C-terminal constructs of the N-terminal 140 amino acid sequence (i.e., GFP-*At*ALDH10A8-140 and *At*ALDH10A8-140-GFP). GFP-*At*ALDH10A8-140 was cytosolic, whereas *At*ALDH10A8-140-GFP was plastidial in both onion epidermal cells and Arabidopsis protoplasts (Fig. 3). Furthermore, the total amount of *At*ALDH10A8-GFP was not increased by the proteasomal inhibitor, MG132 (Supplementary Fig. S5), suggesting that ubiquitin/proteasome-mediated degradation^25^,^26^ was not a factor in our failure to detect *At*ALDH10A8 localization in the plastid. Thus, it can be concluded that: *i*) *At*ALDH10A8 is localized generally in plastids, rather than specifically in leucoplasts, as proposed by Missihoun *et al*.^11^: *ii*) *At*ALDH10A8 possesses a functional plastid targeting signal in its N-terminal region, which is masked by an N-terminal GFP tag; and *iii*) *At*ALDH10A8 must be triggered or changed post-translationally in some manner to facilitate translocation into the plastid. This conclusion is consistent with the marked difference between the slightly acidic pH of cytosol and the moderately alkaline pH optimum for *At*ALDH10A8 activity and for the stromal compartment of plastids.

**Figure 6:**
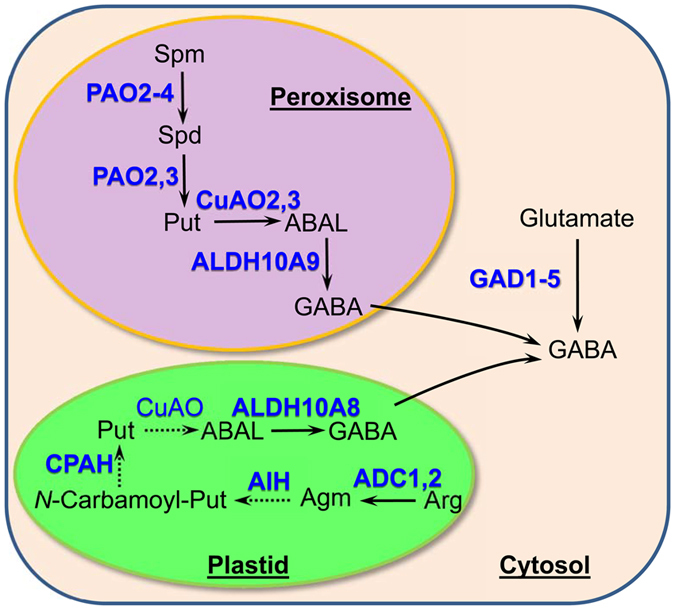
Schematic diagram depicting three subcellular compartments for GABA synthesis in Arabidopsis. Enzymes are shown in blue letters, with biochemically characterized enzymes indicated by bold lettering. Dashed arrows indicates enzymes for which subcellular localization has not been studied. *In silico* analysis predicts three putative CuAOs (At1g31670, At1g31710 and At4g12290) are plastidial (A score of 5 was allocated to these three CuAOs using WoLF PSORT online software). Arabidopsis ADC1 (At2g16500), ADC2 (At4g34710), AIH (At5g08170) and CPAH (At2g27450) have been extensively characterized^43^,^44^,^45^. Abbreviations: ABAL, 4-aminobutanal; ADC, arginine decarboxylase; ALDH, aldehyde dehydrogenase; Agm, agmatine; AIH, agmatine iminohydrolase; Arg, arginine; Carbamoyl-Put, *N*-carbamoylputrescine; CPAH, N-carbamoylputrescine amidohydrolase; CuAO, copper amine oxidase; GABA, 4-aminobutrate; GAD, glutamate decarboxylase; PAO, polyamine oxidase; Put, putrescine; Spd, spermidine; Spm, spermine.

Polyamine oxidation is a potential source of GABA and β-alanine^5^. In peroxisomes, *At*PAO2-4 catalyse the back-conversion of spermine to putrescine, resulting in the production of APAL^8^ (Fig. 6). *At*CuAO2,3 catalyse the terminal oxidation of putrescine to 4-aminobutanal^6^. Here, we demonstrated that peroxisomal *At*ALDH10A9 catalyzes the final step of putrescine oxidation by converting ABAL to GABA in the peroxisome. Putrescine may also be synthesized in Arabidopsis plastids from arginine, although the subcellular localization of agmatine imidohydrolase and *N*-carbamoylputrescine amidohydrolase is unknown^27^. To date, Arabidopsis CuAOs have not been localized to the plastid; however, *in silico* analysis predicts several of them to be plastidial, and they may provide ABAL for conversion to GABA via *At*ALDH10A8 activity.

In the present study, both *in vitro* and *in planta* evidence support the involvement of *At*ALDH10A8 and *At*ALDH10A9 and putrescine-derived GABA in the response to salinity (Figs 4 and 5). Previous *in planta* findings are somewhat contradictory, with the growth of *AtA*LDH10A8 T-DNA knockout mutants (*ko8-2*) being oversensitive to salinity^11^, and Arabidopsis plants overexpressing both *At*ALDH108 and *At*ALDH109 being oversensitive to salinity^28^. In the present study, GABA levels in shoots of WT plants were induced three-fold by treatment with 150 mM NaCl for 2 d, whereas Renault *et al*. (2011) found a 15-fold induction in shoots of 14-day-old WT plants treated with 150 mM NaCl for 4 d, although GABA levels in the untreated plants were similar to those in our control plants. Furthermore, our research with the single *ataldh10a* mutants revealed a reduction in the accumulation of GABA that typically occurs with salinity stress^29^, and reduction would be predicted to be greater in an *aldh10a8/aldh10a9* double mutant. Our study is consistent with the previous suggestion that polyamine-derived GABA functions in the response of soybean roots to salinity stress^30^. Perhaps surprisingly, and considering the higher preference of both *At*ALDH10A8 and *At*ALDH10A9 for APAL over ABAL, the concentrations of β-alanine did not differ among the WT and *ataldh10a8* and *ataldh10a9* mutants without or with salinity stress (Fig. 5). These findings suggest that APAL production from the back-conversion of spermidine and spermine or the catabolism of 1,3-diaminopropane was not enhanced under the salinity stress conditions used in this study. The compartmentation of the two *At*ALDH10s in Arabidopsis, as well as availability of alternative substrates, would be important determinants in the functional role of the enzyme *in vivo*.

## Materials and Methods

### Gene constructs

Two Arabidopsis AMADHs, *ALDH10A8* (GenBank Acc. No. AY093071) and *ALDH10A9* (GenBank Acc. No. AF370333), were extracted from The Arabidopsis Information Resources (TAIR) database with At1g74920 and At3g48170 loci numbers. The *AtALDH10A8* ORF was amplified with *Nde*I and *BamH*I restriction sites using primers CT-F65C and CT-R65 (see Supplementary Table S2 for all primer sequences used in this study), and the *AtALDH10A9* ORF was amplified using primers CT-F66C and CT-R66. Both ORFs were subcloned into the pET15b expression vector (Novagen).

Two different GFP-fusion constructs were prepared for *At*ALDH10A8, *At*ALDH10A9 and *At*ALDH10A8140 (contains the N-terminal 140 amino acids only) to assess their subcellular localization, one with GFP at the N-terminus and another with GFP at the C-terminus. The *AtALDH10A8* ORF was amplified with *BamH*I restriction sites for subcloning into pRTL2∆NS/mGFP-MCS^31^ using primers CT-F65 and CT-R65 to produce the N-terminal GFP fusion construct. The same strategy was used for the *AtALDH10A9* ORF with primers CT-F66 and CT-R66 and *AtALDH10A8-140* with CT-F65 and GFP-A8140R primers. The C-terminal GFP fusion constructs were prepared by amplifying the *AtALDH10A8* ORF with *BamHI* restriction sites using primers CT-F65 and CT-R65B. Similarly, the *AtALDH10A9* and *AtALDH10A8-140* ORFs were amplified with CT-F66 and CT-R66B, and CT-F65 and A8140R-GFP, respectively. The digested products were subcloned into pUC18/BamHI-mGFP^32^ to generate GFP C-terminal fusion proteins.

### Expression and purification of recombinant proteins

The recombinant *Arabidopsis thaliana* [L.] Heynh. ALDH10s were induced in transformed *Escherichia coli* strain BL21 cells (EMD Millipore) by the addition of isopropyl β-D-1-thiogalactopyranoside (final concentration of 0.4 mM) to the Lysogeny broth medium containing 50 μg mL^−1^ ampicillin when the cell culture had an OD_600_ of 0.5. The cells were collected 4 h after induction by centrifugation at 5000× g for 5 min and then stored at −80 °C. Bacterial lysis and purification of the recombinant protein by affinity chromatography were conducted as described previously^7^. High-protein fractions were combined and precipitated on ice by slowly adding solid ammonium sulfate, with gentle stirring, to 80% saturation. Aliquots of precipitated protein were stored at −80 °C. Total proteins were separated by SDS-PAGE gel electrophoresis and visiualized by staining with Coomassie Blue R-250 using standard protocols^33^. Immunoblot analysis was based on a semi-dry method using a mouse monoclonal IgG against the His tag (Santa Cruz Biotechnology, 1:1000) and an anti-mouse IgG–Alkaline Phosphatase (Sigma 1:10000) as primary and secondary antibodies, respectively. Bio-Rad Alkaline Phosphatase Conjugate Substrate Kit was used to detect fusion proteins.

### Determination of kinetic parameters

Enzymatic activity was assayed spectrophotometrically at 340 nm as the reduction of NAD^+^ to NADH^13^ at room temperature in a 250-μL reaction volume using a 96-well microplate. The standard reaction contained 30 mM Bis-tris propane buffer, 30 mM 3-(Cyclohexylamino)-1-propanesulfonic acid buffer at pH 8.5 or 9.5, 0.1 or 0.5 mM NAD^+^ (for *At*ALDH10A8 and *At*ALDH10A9, respectively), 10% glycerol (v/v), 10 mM 2-mercaptoethanol and various concentrations of ABAL, APAL or BAL. No activity was detected in the absence of protein or substrate. A similar buffer mix was used to determine the pH optimum using 7 or 30 μM APAL and 0.1 or 0.5 mM NAD^+^ for *At*ALDH10A8 and *At*ALDH10A9, respectively. For all enzymatic assays, the protein concentrations used (10 nM for APAL and 50 nM for ABAL) were within the linear range. When BAL was used as substrate, 100 nM protein was necessary to obtain the initial rate in the detectable range. The amount of protein was determined via the BioRad protein assay^34^. When NAD^+^ was varied, APAL was adjusted to 7 or 30 μM and ABAL to 40 or 900 μM for *At*ALDH10A8 and *At*ALDH10A9, respectively. Free ABAL and APAL were prepared from 4-aminobutanal diethylacetal and 1-amino-3,3-diethoxypropane, respectively, by boiling with 0.1 M HCl for 10 min in a screwed cap tubes as described elsewhere^35^. The prepared ABAL and APAL were used in assays within 6 h. The pH of the hydrolysate was adjusted to neutrality with KOH just before use. The enzyme reaction was initiated by adding the protein extract. To calculate enzymatic activity, the extinction coefficient for NAD(P)H ɛ_340_ = 6.22 mM^−1^ cm^−1^ was used. Kinetic parameters were determined from the best fit of the initial rates for three independent enzyme preparations (determined as the mean of four technical replicates at the various substrate levels) to the appropriate Michaelis–Menten equation using non-linear regression (SigmaPlot2000, version 12.3; Enzyme Kinetics Module; Systat Software Inc., Point Richmond, CA, USA).

Enzymatic activity was also verified as the ABAL- and APAL-dependent production of GABA and β-alanine, respectively, over a 30-min time course in the presence of NAD^+^ as described previously^16^. For the *At*ALDH10A8 assay, 20 μM APAL or 50 μM ABAL and 0.1 mM NAD^+^ were used, whereas the *At*ALDH10A9 assay contained 50 μM APAL or 800 μM ABAL and 0.5 mM NAD^+^. The remaining reaction components were similar to the standard reaction. The enzymatic reaction was initiated by the addition of 10 nM or 50 nM recombinant protein (for APAL or ABAL, respectively) in a 5-ml final volume. Each reaction was conducted in triplicate using 50 mL test tubes incubated with mild shaking at room temperature. The reactions were terminated by removing 250-μL aliquots at various times and adding them to sulfosalicylic acid (final concentration of 31.1 mg mL^−1^) in microfuge tubes. After centrifugation, the supernatant was neutralized and then filtered. Five microlitres of the supernatant was used for analysis of GABA and β-alanine levels by reverse-phase high performance liquid chromatography after automatic derivatization with *o*-phthalaldehyde as described elsewhere^36^. The retention times for β-alanine and GABA were 9.3 and 10.3 min, respectively.

### Transformation of Arabidopsis cell suspension protoplasts and onion cells

N- and C- terminal GFP-fusion plasmids containing one of the Arabidopsis ALDH10s co-transformed with one of mCherry peroxisomal (pRTL2/Cherry-PTS1)^37^, RFP cytosolic (pRTL2-MCS-RFP-stop)^31^ or RFP plastidial^38^ markers. A ratio of 8:2 (μg GFP:mCherry or RFP) was used for the two plasmid constructs. Protoplasts were prepared from Arabidopsis cell suspension culture ecotype Col-0 as described previously^39^. Polyethylene glycol 4000 (Sigma, 81240) was utilized for protoplast transformation as described previously^40^,^41^,^42^ with some minor modifications. Also, onion epidermal segments were peeled and placed on an MS plate (inner side up) for transient co-transformation using a biolistic particle delivery system (BioRad Laboratories). Onion cells or Arabidopsis protoplasts were incubated for 16 h in the dark at 24 °C prior to microscopy and then examined using an upright Leica DM 6000B confocal laser scanning microscope connected to a Leica TCS SP5 system. Argon and HeNe 542 lasers were activated for visualization of GFP and RFP or mCherry fluorescent proteins. The excitation/emission scan settings were 488 nm/505 nm for the mGFP channel and 543 nm/610 nm for the RFP/mcherry channel. Modulation of laser light intensity and time-lapse scanning were performed using the Leica software LAS AF.

### Plant materials, growth conditions and treatments

All WT and *aldh* T-DNA mutant lines of *Arabidopsis thaliana* were in the Col-0 genetic background. T-DNA insertion lines *aldh10A8-1, aldh10A8-2* and *aldh10A9* (SK24056, Salk079882 and CS822971, respectively) were obtained from The Arabidopsis Biological Resource Center. Seedlings from those seed batches were screened to identify plants containing homozygous T-DNA inserts. Homozygous plants of the *aldh108-1* line were detected with primers SK-10A8-RP and SK-10A8-LP (Table S2) for the WT allele and with SK-10A8-RP and SK-LB for the T-DNA insert, whereas homozygous plants of the *aldh10A8-2* line were detected with primers SALK10A8B-RP and SALK10A8B-LP for the WT allele and SALK10A8B-RP and LBb1.3 for the T-DNA insert. Similarly, homozygous *aldh10A9* plants were detected by with primers SAIL10A9-RP and SAIL10A9-LP for the WT allele and SAIL10A9-RP and LB3 for the T-DNA insert. PCR results are presented in Supplementary Figure S6. PCR primers were designed using the SALK iSECT tool online software. Seeds of homozygous plants were collected and stored for further experiments.

Seeds were surface sterilized in a closed desiccator with chlorine gas for 3 h (http://www.plantpath.wisc.edu/fac/afb/vapster.html), and transferred to plates containing half strength MS medium with 0.6% agar. These plates were subjected to a stratification regime (3 d, 4 °C) and then placed in a tissue culture chamber (22 °C, 14 h light/18 °C, 10 h dark, light intensity of 60 μmol m^−2^ s^−1^) for 8 d. To assess the impact of salinity on root growth, three WT and mutant seedlings were transferred to a single 14-cm plate containing half strength MS medium supplemented with 0, 100, 150 or 200 mM NaCl and the initial length of the primary root apex was noted on the exterior surface of the plate. The plate was stored vertically and photographed 4 d after transfer, and then the new growth was estimated using ImageJ software (http://rsbweb.nih.gov/ij/). The experimental design was completely randomized and comprised of at least three plate replicates for each treatment.

To assess the impact of salinity on GABA and β-alanine levels, individual 12-d-old WT or *aldh* plants were transferred from plates to 7-cm square pots containing LC1 soil mix (Sungrow, Canada) and placed in a growth chamber (22 °C, 11 h light/18 °C, 13 h dark, light intensity of 220 μmol m^−2^ s^−1^, RH of 50%). At 5 weeks of age, 150 ml of 150 mM NaCl solution or water was added to the top of each pot, so that the shoot was submerged for approximately 10 s. Shoots of treated and non-treated plants were individually and simultaneously harvested 48 h later, immediately flash frozen in liquid N_2_, and stored at −80 °C. Each sample was ground with a cold mortar and pestle and 150 mg of the powder was added to 750 μl of sulfosalicylic acid (31.1 mg mL^−1^) in a microfuge tube and vortexed for 10 min. After centrifugation, the supernatant was transferred into another tube, neutralized with 4 M KOH, and passed through a 0.45 μm filter before storage at −20 °C for no longer than 10 d before HPLC analysis as described above.

## Additional Information

**How to cite this article**: Zarei, A. *et al*. Arabidopsis aldehyde dehydrogenase 10 family members confer salt tolerance through putrescine-derived 4-aminobutyrate (GABA) production. *Sci. Rep.* **6**, 35115; doi: 10.1038/srep35115 (2016).

## Supplementary Material

Supplementary Information

## Figures and Tables

**Table 1:** Kinetic parameters for *At*ALDH10A8 and *At*ALDH10A9^a^.

Substrate/coenzyme	*V*_max_^b^	*At*ALDH10A8			*V*_max_	*At*ALDH10A9		
		*K*_m_	*K*_is_	*k*_cat_*/K*_m_		*K*_m_	*K*_is_	*k*_cat_*/K*_m_
ABAL	1.6 ± 0.3	25.1 ± 2.5	55.7 ± 26.0	0.06	9.5 ± 1.5	460 ± 65	2.6E03 ± 7.5E02	0.02
NAD^+^	0.8 ± 0.1	20.8 ± 3.0	1.2E06 ± 6.4E05	0.04	4.2 ± 0.5	73.6 ± 16.4	9.5E06 ± 1.5E06	0.06
APAL	15.8 ± 1.4	14.4 ± 2.7	5.5 ± 0.3	1.03	15 ± 3.3	17.3 ± 4.1	1.9E04 ± 5.8E03	0.82
NAD^+^	3.6 ± 0.6	13.2 ± 1.5	5.4E05 ± 5.3E03	0.26	13.4 ± 3	86 ± 16	6.1E06 ± 3E05	0.15

Initial rate of NAD^+^ reduction was plotted against increasing ABAL, APAL or NAD^+^ concentration and the data were fit to the appropriate Michaelis-Menten equation. pH of assays were adjusted to 8.5 and 9.5 for *At*ALDH10A8 and *At*ALDH10A9 respectively. Each value represents the mean (± SE) of three enzyme preparations.

^a^NAD^+^ dependence was estimated in the presence of 7 and 30 μM APAL and 40 and 900 μM ABAL for *At*ALDH10A8 and *At*ALDH10A9, respectively to minimize substrate inhibition; therefore, the values in this table are apparent. Similarly, ABAL and APAL dependence assays were measured at 100 μM NAD^+^ for *At*ALDH10A8 and 500 μM NAD^+^ for *At*ALDH10A9. All kinetic curves are shown in Supplementary Figure S4.

^b^*V*_max_ (μmol min^−1^ mg^−1^ protein), *K*_m_ (μM), catalytic efficiency (*k*_cat_/*K*_m_, μM^−1^ s^−1^) and substrate inhibition constant (*K*_is,_ μM) are shown for each substrate or coenzyme.
