# Corrigendum: Arabidopsis aldehyde dehydrogenase 10 family members confer salt tolerance through putrescine-derived 4-aminobutyrate (GABA) production

**DOI:** 10.1038/srep46967

**Published:** 2018-04-04

**Authors:** Adel Zarei, Christopher P. Trobacher, Barry J. Shelp

Scientific Reports 6: Article number: 35115; 10.1038/srep35115 published online: 10 11 2016; updated: 04 04 2018.

This Article contains errors.

One of the T-DNA insertion lines was mislabelled. *aldh10A8-2* mutant line used in the study was incorrectly labelled as Salk 079882. The correct line number is Salk 079892. Therefore, all instances of “Salk 079882” should read “Salk 079892”.

In the Results section, the subheading,

“AtALDH10A8 and AtALDH10A9 localized to peroxisome and plastid”

should read:

“AtALDH10A8 and AtALDH10A9 localized to plastid and peroxisome”

In the Discussion section,

“Missihoun *et al.*^11^ has also reported that AtALDH10A8 is cytosolic; however, its N-terminal portion (1–140 aa fused to GFP) apparently targets the leucoplast, rather than the chloroplast, although organelle markers were not used.”

should read:

“Missihoun *et al.*^11^ has also reported that GFP-AtALDH10A8 is cytosolic; however, its N-terminal portion (1–140 aa fused to GFP) apparently targets the leucoplast, rather than the chloroplast, although organelle markers were not used.”

These changes do not affect the conclusions of the Article.

