# Supplementary material for: Arabidopsis aldehyde dehydrogenase 10 family members confer salt tolerance through putrescine-derived 4-aminobutyrate (GABA) production
Source: Sci Rep. 2016 Oct 11;6:35115. doi: 10.1038/srep35115 (PMC5057122; doi:10.1038/srep35115)
Supplement: Supplementary Information [file srep35115-s1.pdf]

Arabidopsis aldehyde dehydrogenase 10 family members confer salt tolerance through putrescine-derived 4-aminobutyrate (GABA) production

Adel Zarei[1], Christopher P. Trobacher[1, 2], Barry J. Shelp[1]

[1]Department of Plant Agriculture, University of Guelph, Guelph, Ontario, Canada N1G 2W.

[2]Present address: NutriAg Ltd., 62 Arrow Rd, North York, Ontario, Canada M9M 2L8.

Correspondence and request for materials should be addressed to B. J. S. (E-mail address: bshelp@uoguelph.ca).

Supplementary material

Table S1. Kinetic parameters of *At*ALDH10A8 and *At*ALDH10A9 at various pHs. Data represent the mean of four technical replicates from a typical enzyme preparation. V_{max} (µmol min^{-1} mg^{-1} protein), K_m (µM), catalytic efficiency (k_{cat}/K_{m}, µM^{-1} s^{-1}) and substrate inhibition constant (K_{is}, µM) are shown for APAL as the substrate.

Enzyme	pH	K_m	V_{max}	K_{is}	k_{cat}/K_m
*At*ALDH10A8	8.5	10.7	13.6	5	1.19
	9.5	27.6	27.1	3	0.92
	10.5	25.4	24.6	10	0.91
*At*ALDH10A9	8.5	21.0	12.4	2300	0.55
	9.5	25.5	20.6	2472	0.76

Table S2. **List of primers employed for gene cloning and molecular analysis of Arabidopsis ALDH mutants.**

Primer	Sequence
CT-F65C	ccatatggcgattccgatgcctactcg
CT-R65	cggatccttagttgggagatttgtaccatccccagg
CT-F66C	ccatatggcgattacggtgccgagacgg
CT-R66	cggatcctcagagcttggaaggaggtttgtaccatcc
CT-F65	cggatccatggcgattccgatgcctactcg
CT-R65	cggatccttagttgggagatttgtaccatccccagg
CT-F66	cggatccatggcgattacggtgccgagacg
CT-R66	cggatcctcagagcttggaaggaggtttgtaccatcc
CT-F65	cggatccatggcgattccgatgcctactcg
CT-R65B	cggatccgttgggagatttgtaccatccccaggg
CT-F66	aggatccatggcgattacggtgccgagacg
CT-R66B	cggatccgagcttggaaggaggtttgtaccatcccc
GFP-A8140R	gcggatccttacatgggaagcgagactggtg
A8140R-GFP	gcggatcccatgggaagcgagactggtg
SK-10A8-RP	ttatctcaacggccaagagtg
SK-10A8-LP	ttcttgatactgctgcaccag
SK-LB	atacgacggatcgtaatttgtcg
SALK10A8B-RP	ttgatgctctaaaagctaaacgg
SALK10A8B-LP	tgttttgactggtttcggttc
LBb1.3	attttgccgatttcggaac
SAIL10A9-RP	aatgctttcctagcagcttcc
SAIL10A9-LP	tttattgttccgtggaaatgg
LB3	tagcatctgaatttcataaccaatctcgatacac

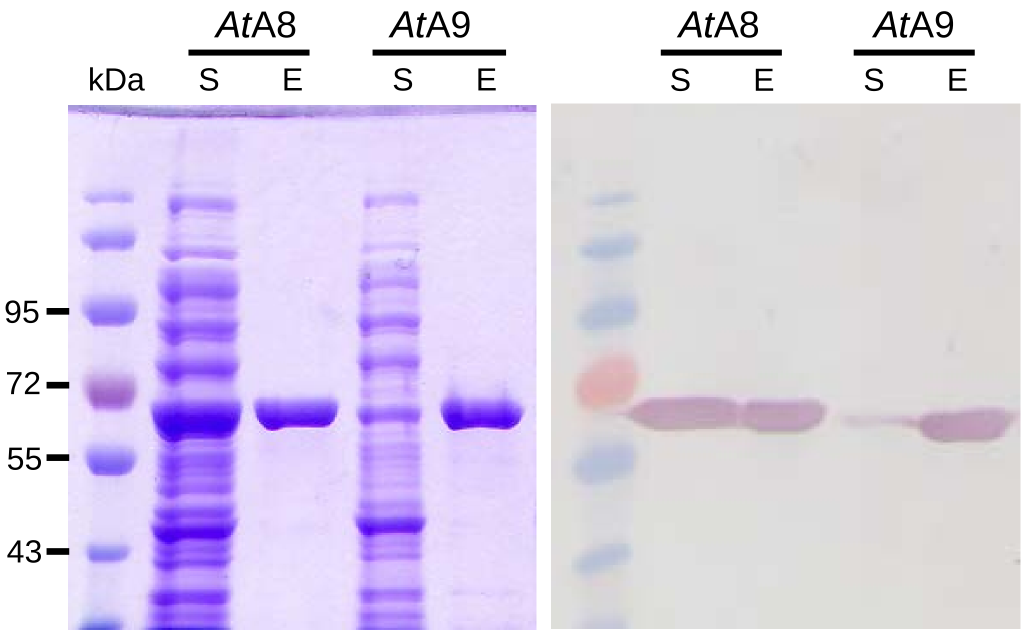

Figure S1. Purification and detection of *At*ALDH10A8 and *At*ALDH10A9. Recombinant proteins were expressed in *E. coli* strain BL21 (DE3) Rosetta pLysS cells and passed through a Ni^{+} column and detected with Coomassie Brilliant Blue (left panel) or with Penta-His HRP conjugate following immunoblot analysis (right panel). Protein size markers are indicated by kDa. S and E represent soluble and affinity-purified fractions. Labels *At*A8 and *At*A9 represent *At*ALDH10A8 and *At*ALDH10A9 respectively.

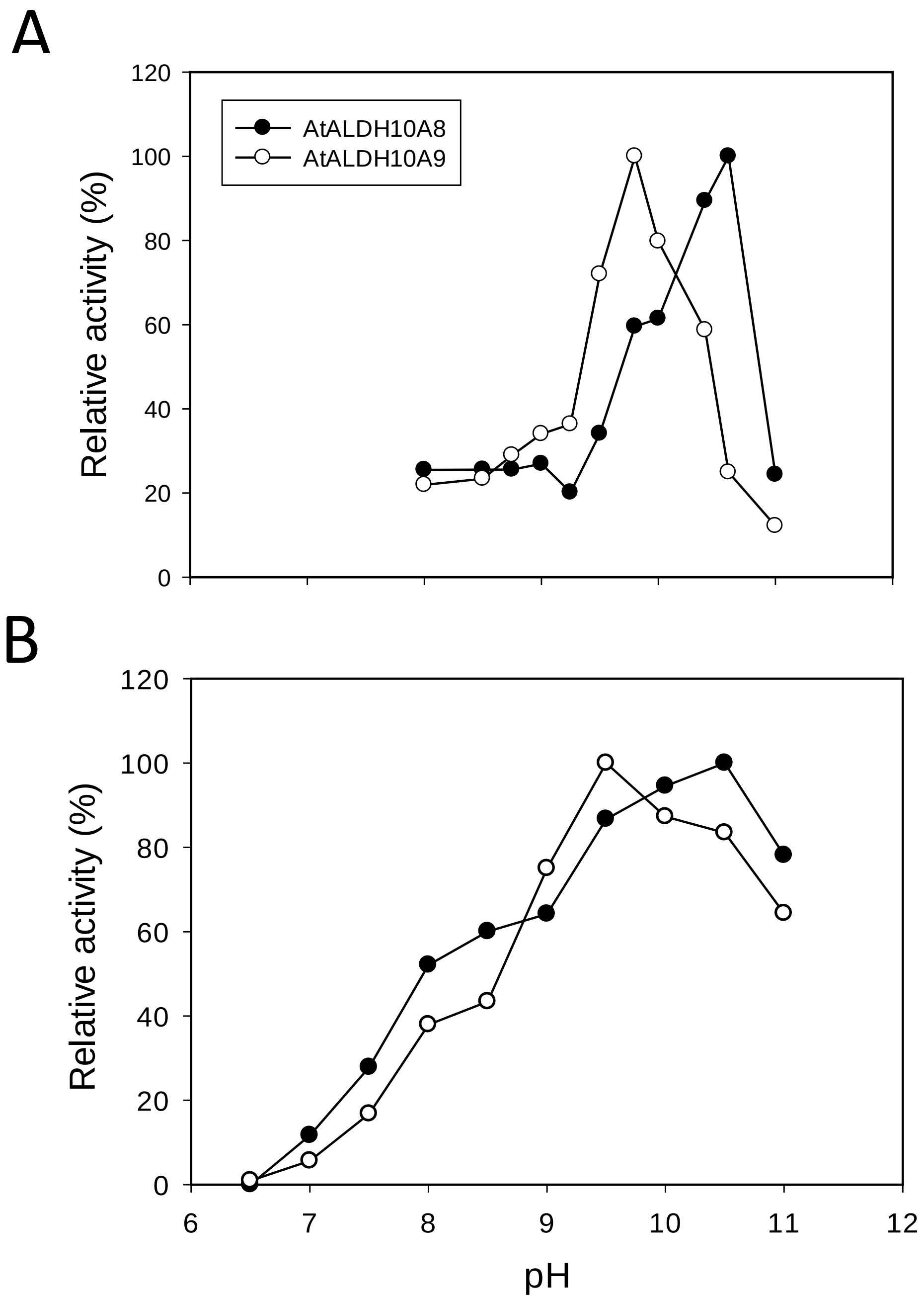

Figure S2. Relative activity of *At*ALDH10A8 and *At*ALDH10A9 as a function of pH. (A) Assays were conducted at saturating concentrations of substrate and co-factor (1 mM ABAL and 1 mM NAD^+). Activities are expressed as a percentage of the corresponding maximum rate (100%: 0.9, 5.2 µmol $min^{-1}mg^{-1}$ protein for *At*ALDH10A8 and *At*ALDH10A9, respectively). **(B)** Assays were determined using subsaturating concentrations of APAL (7 and 30 µM for *At*ALDH10A8 and *At*ALDH10A9, respectively), NAD^+ (100 and 500 µM for *At*ALDH10A8 and

*At*ALDH10A9, respectively) and a mixture of two buffers at room temperature. Activities are expressed as a percentage of the corresponding maximum rate (5 and 8.2 µmol min^{-1} mg^{-1} protein for *At*ALDH10A8 and *At*ALDH10A9, respectively).

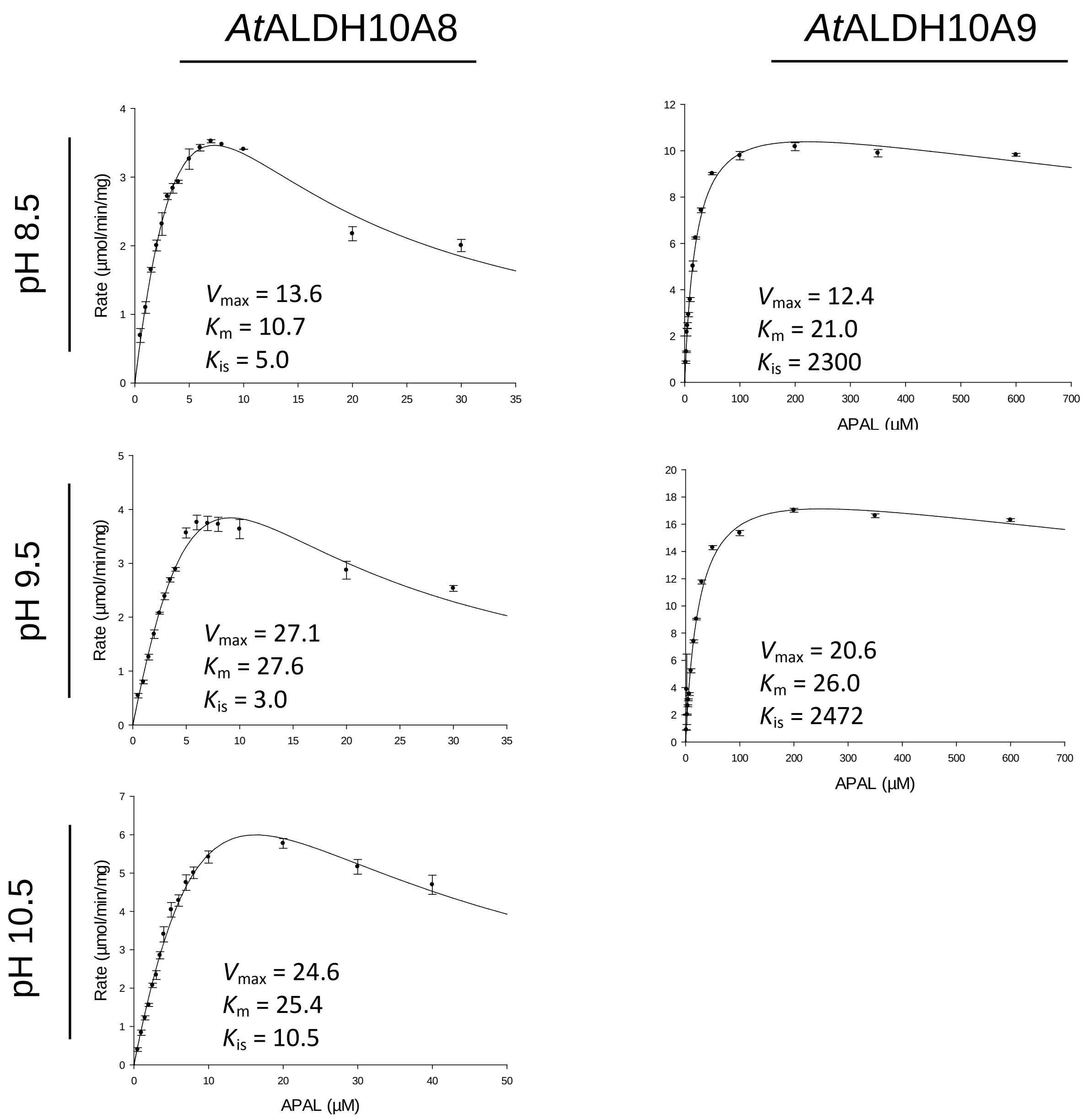

Figure S3. Kinetic characterization of recombinant *At*ALDH10A8 and *At*ALDH10A9 activities at the various pHs. Kinetic data from a single typical enzyme preparation were best fit by the appropriate Michaelis-Menten equation. Each datum represents the mean ± SD of four technical measurements. V_{max} (µmol min^{-1} mg^{-1} protein), K_m (µM), catalytic efficiency ($k_{cat}/K_{m,}$ µM^{-}

^{1} s^{-1}) and substrate inhibition constant ($K_{is,}$ μM) are shown for APAL as the substrate. NAD^+ concentration was 0.1 mM and 0.5 mM for *At*ALDH10A8 and *At*ALDH10A9, respectively.

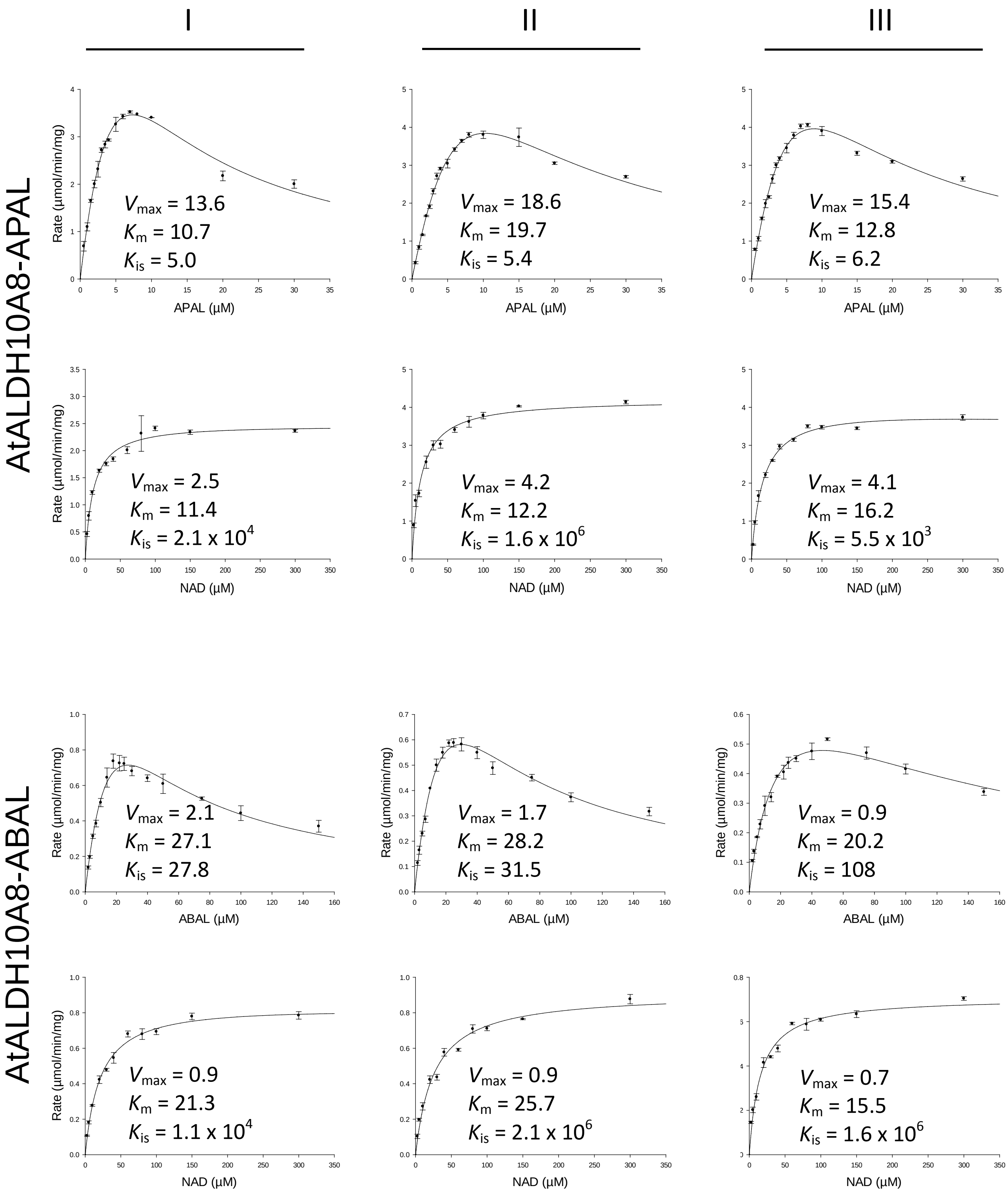
I
II
III
AtALDH10A8-APAL
AtALDH10A8-ABAL
Rate (µmol/min/mg)
APAL (µM)
NAD (µM)
ABAL (µM)
V_{max} = 13.6
K_m = 10.7
K_{is} = 5.0
V_{max} = 18.6
K_m = 19.7
K_{is} = 5.4
V_{max} = 15.4
K_m = 12.8
K_{is} = 6.2
V_{max} = 2.5
K_m = 11.4
K_{is} = 2.1 x 10^4
V_{max} = 4.2
K_m = 12.2
K_{is} = 1.6 x 10^6
V_{max} = 4.1
K_m = 16.2
K_{is} = 5.5 x 10^3
V_{max} = 2.1
K_m = 27.1
K_{is} = 27.8
V_{max} = 1.7
K_m = 28.2
K_{is} = 31.5
V_{max} = 0.9
K_m = 20.2
K_{is} = 108
V_{max} = 0.9
K_m = 21.3
K_{is} = 1.1 x 10^4
V_{max} = 0.9
K_m = 25.7
K_{is} = 2.1 x 10^6
V_{max} = 0.7
K_m = 15.5
K_{is} = 1.6 x 10^6

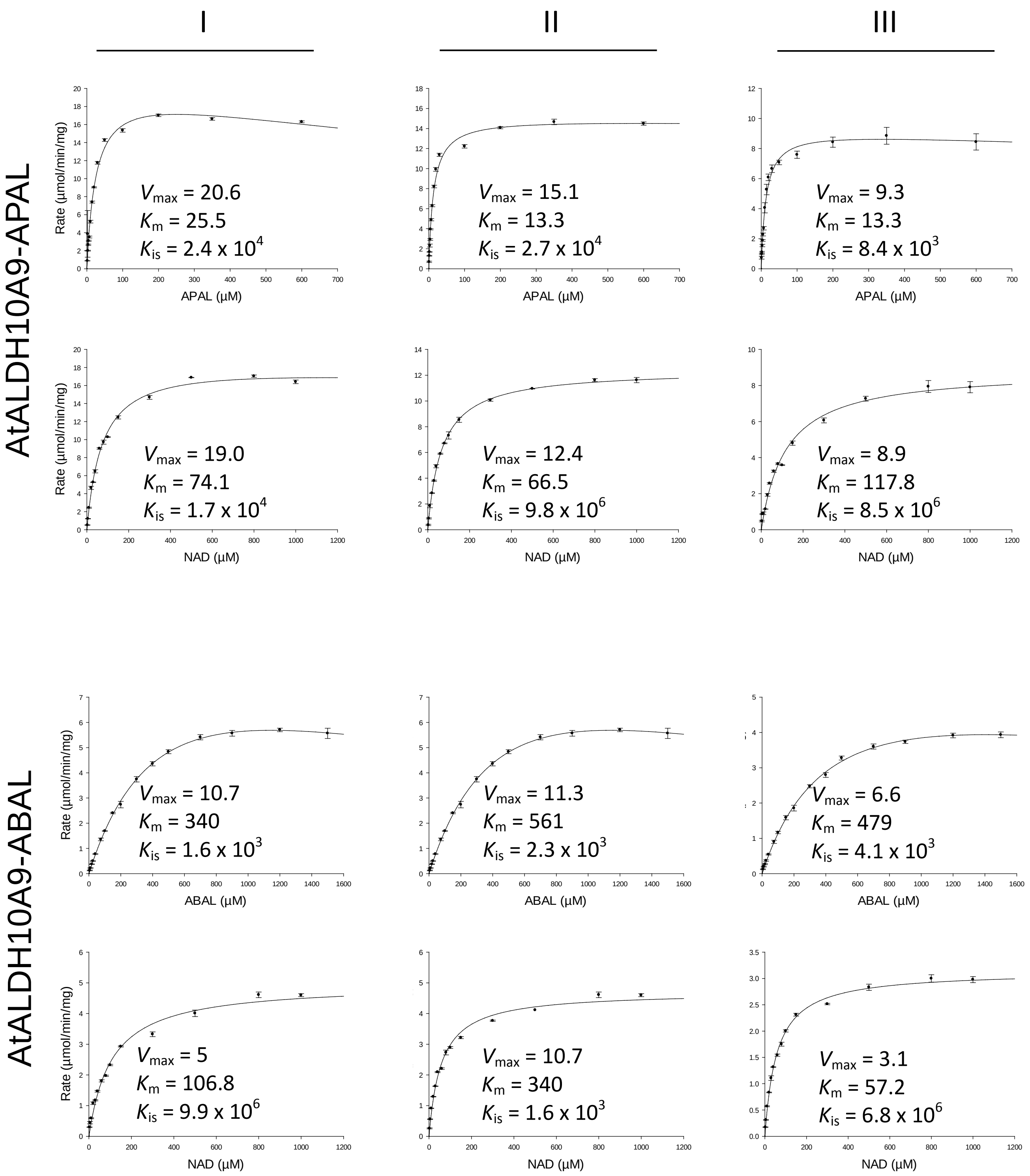

Figure S4. Kinetics of *At*ALDH10A8 and *At*ALDH10A9 with APAL and ABAL as substrates. The initial velocity was recorded as the rate of production of NADH with varying

concentrations of APAL, ABAL or NAD. The column beneath each Roman number represents data obtained from a single typical enzyme preparation and each datum represents the mean of four technical replicates. Data were fit to the appropriate Michaelis-Menten equation and the resulting kinetic parameters are indicated.

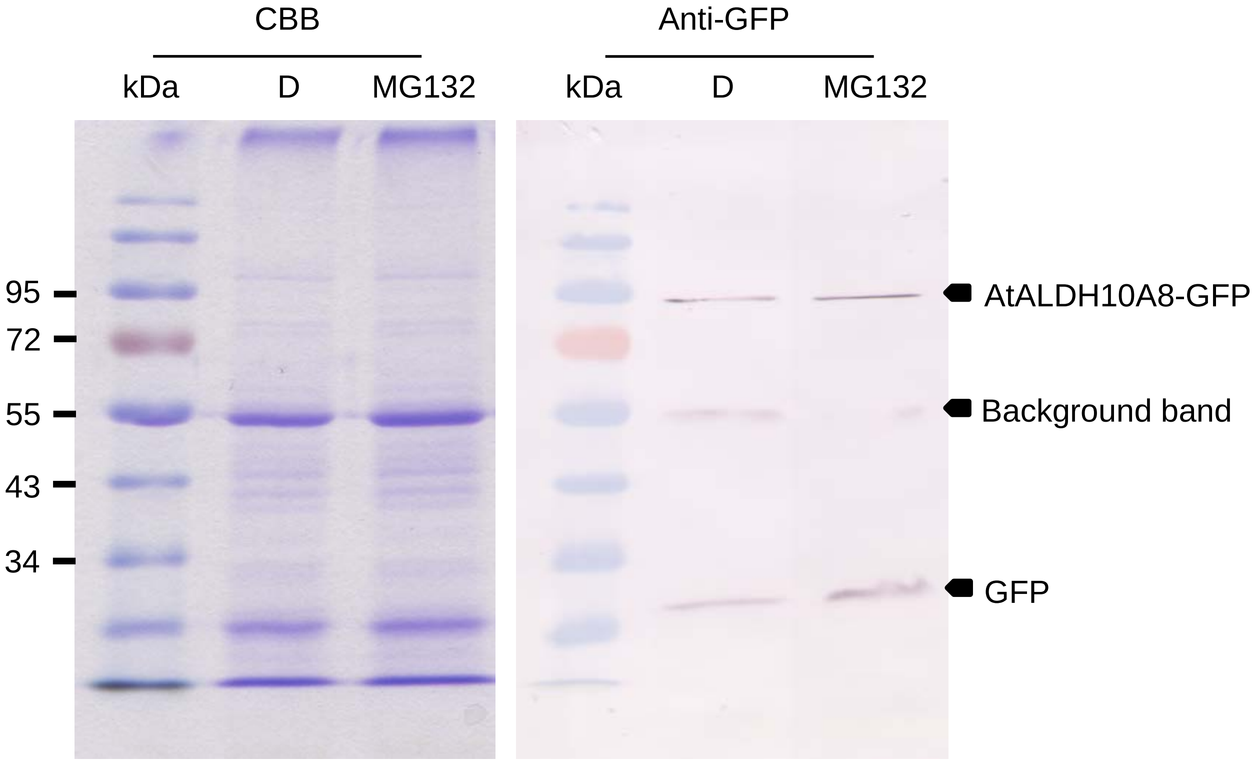

Figure S5. Detection of *in vivo* expressed *At*ALDH10A8-GFP protein using immunoblot analysis. Total protein extract prepared from Arabidopsis protoplasts co-expressing GFP and AtALDH10A8-GFP treated with 100 µM MG132 or DMSO (D) for 4 h was subjected to the anti-GFP antibody. The intensity of the GFP bands on the blot confirm similar transformation efficiency while intensity of total protein bands in the gel stained with Coomassie Brilliant Blue (CBB) shows similar loading of total protein.

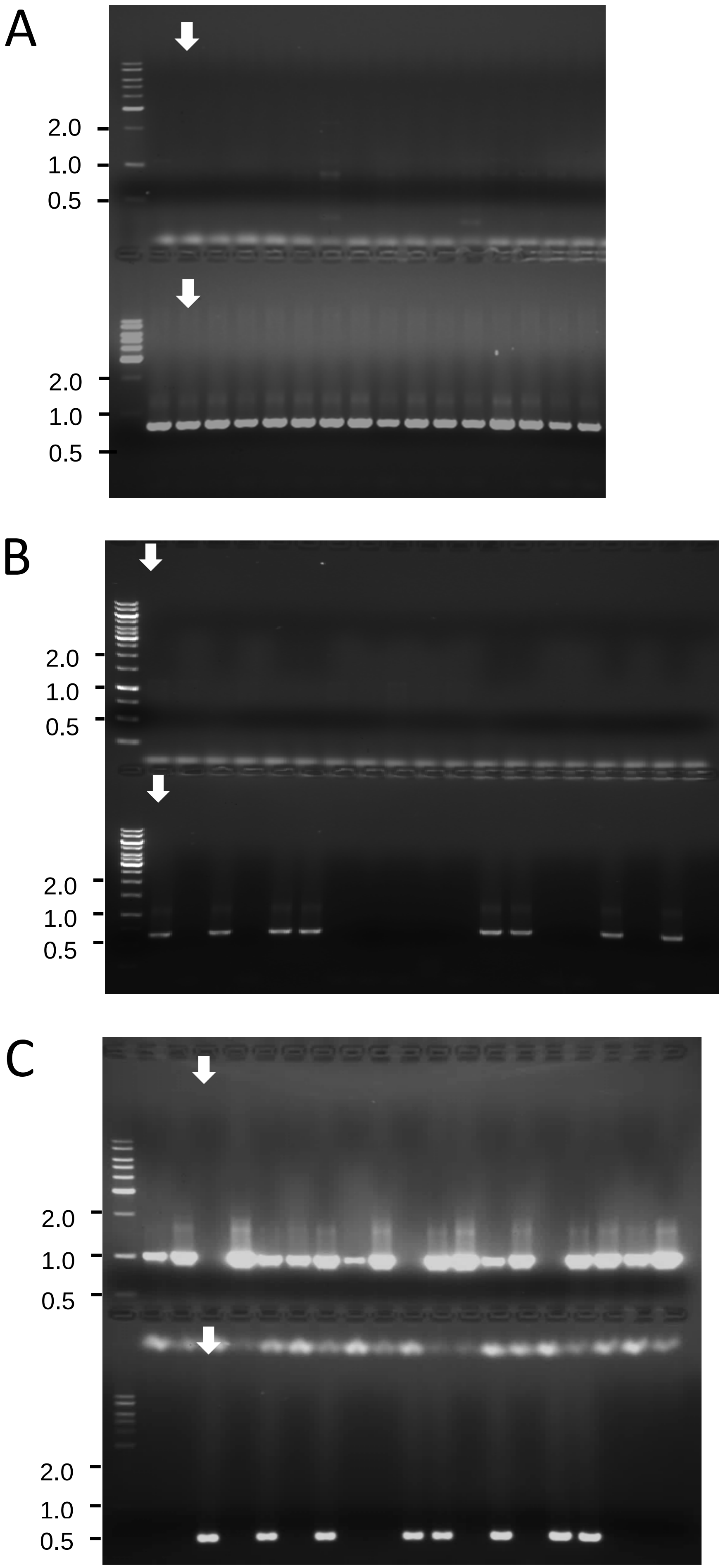
A
2.0
1.0
0.5
2.0
1.0
0.5
B
2.0
1.0
0.5
2.0
1.0
0.5
C
2.0
1.0
0.5
2.0
1.0
0.5

Figure S6. Molecular analysis of *At*ALDH10A mutants. PCR detection of wild-type and T-DNA insertion alleles indicate homozygosity in lines under consideration. **(A)** Genotyping line *aldh10A8-1* (SK24056): Top row shows result of PCR reactions containing specific primers for wild-type alleles, whereas bottom row shows results of PCR reactions primers containing primers specific for the T-DNA insertion allele, giving fragments of 750 bp. **(B)** Genotyping line *aldh10A8-2* (Salk079882): Top row shows result of PCR reactions containing specific primers for wild-type alleles, whereas bottom row shows results of PCR reactions primers containing primers specific for the T-DNA insertion allele, giving fragments of 700 bp. **(C)** Genotyping line *aldh10A9* (CS822971): Top row shows result of PCR reactions containing specific primers for wild-type alleles, whereas bottom row shows results of PCR reactions primers containing primers specific for the T-DNA insertion allele, giving fragments of 450 bp. White arrows indicate the homogygous plant of each line, which was selected for further experimentation.
